# DNA Damage Response Mechanisms in Model Systems

**DOI:** 10.3390/genes14071385

**Published:** 2023-06-30

**Authors:** Jeannine R. LaRocque, Mitch McVey

**Affiliations:** 1 Department of Human Science, Georgetown University, Washington, DC 20057, USA; 2 Department of Biology, Tufts University, Medford, MA 02155, USA; mitch.mcvey@tufts.edu

Cells are constantly assaulted by endogenous and exogenous sources of DNA damage that threaten genome stability. To maintain genome integrity, organisms have evolved a myriad of responses, including transcriptional programs, cell cycle regulation, lesion bypass, and direct repair—collectively called the DNA Damage Response (DDR). While much of the research describing these responses has utilized mammalian cell culture systems, studies performed in model organisms have also provided critical insights into the organismal regulation of damage responses [1,2,3,4,5,6,7]. Given the conservation of the damage response and repair proteins across various species, model organisms continue to provide meaningful insight into the mechanisms of responding to and repairing DNA damage. Additionally, model organisms provide the opportunity to employ classical genetic techniques to elucidate the factors that contribute to the DDR. This Special Issue includes both original research papers and review articles that highlight discoveries made in the areas of DNA repair and damage responses, with an emphasis on findings from eukaryotic model systems. 

Many of the articles in this Special Issue focus on the repair of a particularly toxic type of DNA lesion, the DNA double-strand break (DSB). DSBs are primarily repaired through non-homologous end joining (NHEJ) and homologous recombination (HR). While HR is typically considered error-free due to the use of a homologous sequence to repair the event, NHEJ can be error prone due to the insertions and deletions of nucleotides at the break site. The choice between these two pathways is dictated by several factors, including phase of the cell cycle and genomic location of the break. The eukaryotic genome is packaged into different domains that impact nuclear structure, gene expression, and DNA DSB pathway choice. Of note is silenced chromatin, which includes heterochromatin. Heterochromatin domains are typically transcriptionally inert and often contain repetitive DNA. Two review articles in this issue synthesize current understanding of the response to DSBs within silenced chromatin. These genomic domains require a unique response to DNA damage, which is described by Kendek et al. in a review describing the preference for DSB repair pathway choice and post-translational modifications related to the DDR in heterochromatin [8]. The recent expansion of work in this field has been made possible by the development of novel tools and assays to study heterochromatin repair in model systems, which are highlighted by Rawal and colleagues [9].

In addition to genome structure, the cell cycle also contributes to regulating the DNA damage response. In their review, Clay and colleagues describe how model organisms have shaped our understanding of the DDR, in particular within the context of the cell cycle [10]. They discuss the conservation of the DDR in both mitosis and meiosis across different organisms. Importantly, they also highlight the unique features of each system, providing rationale for the use of model systems to answer DDR-related questions.

Lastly, Shen et al. provide a review focusing on the use of plants as a model system [11]. Genetic experiments requiring manipulation of the plant genome are often achieved by *Agrobacterium*-mediated transformation (AMT) of T-DNA and gene targeting (GT), both of which require DNA DSB formation. Practically, these lesions can lead to cell death if unrepaired or genome rearrangements if repaired incorrectly. Thus, understanding the mechanisms that regulate the repair of these DSBs in plants is critical.

The original research articles featured in this Special Issue focus on new findings related to DNA damage repair in *Drosophila melanogaster.* Several unique features in *Drosophila* allow experimental manipulations in this model organism to address specific questions regarding DSB repair pathway choice. For example, Bergerson et al. aimed to address whether the increased incidence of cancer in long-shift workers was associated with a shift from repair of DNA DSBs from a more error-free HR to error-prone NHEJ. They utilized the ability to disrupt circadian rhythms in *Drosophila* by experimentally shifting the light/dark cycle and measuring the repair outcome of a DNA DSB reporter assay [12]. This study found that shifting light/dark cycles did not result in an increase in NHEJ repair, although they noted that other factors (such a genomic context [8]) may be sensitive to shifts in circadian rhythms resulting in changes in repair outcome.

In addition to applications of experimental manipulations, non-murine model organisms also play an integral role in determining the function of proteins that may be essential in mammals. For example, null mutations in mammalian C-terminal Interaction Protein (CtIP) are lethal, thus preventing classical reverse genetics experiments; however, *Drosophila* with *CtIP* deletions are viable [13]. Yannuzzi et al. demonstrated that *Drosophila* CtIP is involved in homology-directed repair using DSB reporter assays that measure both HR and single-strand annealing (SSA), a third DSB repair mechanism that is homology-directed and can require extensive end resection. This work demonstrated a decrease in HR as well as “short” (500 bp) and “long” (3 kb) SSA, confirming a role for CtIP in end resection [13].

Using classical genetic techniques in model organisms allows for the ability to determine whether proteins exhibit epistasis or function in redundant pathways. This is a common approach using *Drosophila*, which affords the tracking of several mutant alleles in relatively short generation times. Thomas et al. investigated the contributions of a variety of helicases to the repair of DNA double-strand gaps following transposon excision [14]. Analysis of *helq*, *fancm*, and *blm* single and combination mutants suggested that the FANCM and HELQ helicases function upstream of BLM to create recombination intermediates that are then unwound by BLM. Without BLM, these structures are processed in a way that creates large genomic deletions.

Lastly, model organisms can be utilized in forward genetic screens to identify proteins involved in the DDR. Mitchell and colleagues used classical genetic mapping techniques to identify *mutagen sensitive 109* (*mus109*) as the *DNA2* gene, which encodes a helicase/nuclease protein [15]. They characterized *mus109* mutants as sensitive to agents that cause DNA adducts as well as DNA DSBs, thus establishing the first transgenic allele set of *Drosophila DNA2* and, importantly, contributing to our understanding of the conservation of DNA2 across species.

Thus, this Special Issue has demonstrated that model organisms continue to provide essential information about how the eukaryotic DDR maintains the integrity of our genome and reveal insights that would not be possible in cell-based systems.

## Data Availability

Not applicable.
